# LRRK2 mutations in Parkinson's disease: Confirmation of a gender effect in the Italian population

**DOI:** 10.1016/j.parkreldis.2014.04.016

**Published:** 2014-08

**Authors:** Roberto Cilia, Chiara Siri, Damiana Rusconi, Roberta Allegra, Andrea Ghiglietti, Giorgio Sacilotto, Michela Zini, Anna L. Zecchinelli, Rosanna Asselta, Stefano Duga, Anna M. Paganoni, Gianni Pezzoli, Manuela Seia, Stefano Goldwurm

**Affiliations:** a Parkinson Institute, Istituti Clinici di Perfezionamento, via Bignami 1, 20126 Milan, Italy; b Medical Genetics Laboratory, Foundation IRCCS “Ospedale Maggiore Policlinico, Mangiagalli e Regina Elena”, Milan, Italy; c MOX, Dipartimento di Matematica, Politecnico di Milano, Italy; d Dipartimento di Biotecnologie Mediche e Medicina Traslazionale, Università degli Studi di Milano, Italy

**Keywords:** Parkinson disease, Genetics, LRRK2, Gender

## Abstract

**Background:**

The relative risk of developing idiopathic PD is 1.5 times greater in men than in women, but an increased female prevalence in LRRK2-carriers has been described in the Ashkenazi Jewish population. We report an update about the frequency of major LRRK2 mutations in a large series of consecutive patients with Parkinson's disease (PD), including extensive characterization of clinical features. In particular, we investigated gender-related differences in motor and non-motor symptoms in the LRRK2 population.

**Methods:**

2976 unrelated consecutive Italian patients with degenerative Parkinsonism were screened for mutations on exon 41 (G2019S, I2020T) and a subgroup of 1190 patients for mutations on exon 31 (R1441C/G/H). Demographic and clinical features were compared between LRRK2-carriers and non-carriers, and between male and female LRRK2 mutation carriers.

**Results:**

LRRK2 mutations were identified in 40 of 2523 PD patients (1.6%) and not in other primary parkinsonian syndromes. No major clinical differences were found between LRRK2-carriers and non-carriers. We found a novel I2020L missense variant, predicted to be pathogenic. Female gender was more common amongst carriers than non-carriers (57% *vs.* 40%; *p* = 0.01), without any gender-related difference in clinical features. Family history of PD was more common in women in the whole PD group, regardless of their LRRK2 status.

**Conclusions:**

PD patients with LRRK2 mutations are more likely to be women, suggesting a stronger genetic load compared to idiopathic PD. Further studies are needed to elucidate whether there is a different effect of gender on the balance between genetic and environmental factors in the pathogenesis of PD.

## 1 Introduction

Leucine-rich Repeat Kinase 2 (*LRRK2*) is one of the genes that is most frequently involved in Parkinson's disease (PD)[e-1]. Many variants in this gene have been described, but only a few of them are certainly pathogenic, including mutations G2019S and R1441C/G/H. The most common mutation is G2019S, whose frequency varies considerably among populations [e-1]. Penetrance of *LRRK2* mutations is incomplete and age-related [1].

Recent studies have shown that gender distribution is even among Ashkenazi Jews *LRRK2* carriers [2], [3], [4] and in other genetic forms of PD [5]. These studies also suggested that there may be gender-related differences in the balance between genetic and environmental factors, the genetic load being heavier in women than in men [6]. On the other hand, it is still debated whether PD *LRRK2* carriers display a distinctive clinical phenotype compared to idiopathic PD or not [e-1–e-4].

We compared demographic and clinical features between carriers and non-carriers to shed light on the possible impact of *LRRK2* mutations on clinical features and to investigate whether or not *LRRK2* status influences gender distribution and PD phenotype.

## 2 Methods

### 2.1 Subjects

We studied 2976 unrelated consecutive patients with degenerative parkinsonism, who contributed to the ‘Parkinson Institute Biobank’ (http://www.parkinsonbiobank.com) from June-2002 to January-2011. All patients were enrolled consecutively, not selected for any clinical or familial feature. A first group of 1245 patients was described elsewhere [7]. Here, we report an update on 1734 additional patients, reaching a total of 2976 unrelated consecutive patients. All patients were tested for the major *LRRK2* mutation G2019S in exon 41. Exon 31, containing the mutations R1441C/G/H, was analyzed in a subgroup of 1190 patients (a first group of consecutive patients and then only familial cases), out of whom 1088 had PD (428 familial and 660 sporadic cases). In addition, when *LRRK2*-mutations were identified in a proband, we studied all available family members with a definite PD diagnosis. Thus, we enrolled 10 additional living relatives affected by PD and carriers of the G2019S mutation from nine families. Patients found to be carriers of mutations in any other PD-related genes (see Supplementary material) were not excluded from *LRRK2* genetic analysis. However, to minimize confounding effects on the phenotype, we excluded such patients from the comparative analysis of clinical features.

Clinical diagnosis was made according to established diagnostic criteria [e-5]. All the 2976 patients had a diagnosis of primary degenerative parkinsonism: 2523 fulfilled criteria for PD, 53 for Dementia with Lewy Bodies, 128 for Multiple System Atrophy, 14 for Frontotemporal Dementia, 103 for Progressive Supranuclear Palsy, and 33 for Corticobasal Degeneration. In the remaining 122 patients the clinical diagnosis was still uncertain and reported as Undefined Primary Parkinsonism. Among the 2523 PD patients, 1488 were male (59%), mean age at onset was 55.76 years (range 14–87; SD ± 10.94), mean disease duration was 14.12 years (range 2–56; SD ± 6.75).

Family history in LRRK2-carriers was evaluated during formal genetic counseling. Proband relatives with possible parkinsonism not available for neurological examination were assumed to have PD only in case of previous diagnosis and when prescription of dopaminergic therapy was reported. In non-carriers, family history was collected by means of a questionnaire. Formal genetic counseling sessions occurred in most cases when a relative was reported to be affected by PD. Patients were classified as “familial” if at least one among their 1st, 2nd, or 3rd degree relatives had a formal diagnosis of PD.

Clinical features of *LRRK2*-carriers were compared to those of patients whose molecular analysis was negative for the major *LRRK2* mutations and for other mutations in known PD genes. Demographic and clinical data were collected from all patients, including the latest Unified Parkinson's Disease Rating Scale (UPDRS) scores from part I to III in the medication-On and -Off state, and the Hoehn and Yahr stage. Major milestones of PD progression were explored by transforming specific UPDRS items into dichotomous variables, i.e. falls (item 13, score ≥ 2), postural instability (score ≥ 2 for item 30), non-levodopa-responsive freezing of gait (item 14, score ≥ 2), dysphagia (item 7, score ≥ 2) and speech difficulties (item 18, score ≥ 2).

The study was approved by the local Ethics Committee and written informed consent was obtained from all subjects.

### 2.2 Mutation analysis

R1441C/G/H, G2019S and I2020T mutations were analyzed with standard methods (see Supplementary materials). *LRRK2* haplotype was analyzed in all G2019S carriers (Table e-1).

### 2.3 Statistical analysis

We compared demographic and clinical variables between *LRRK2*-carriers and non-carriers, and we also tested gender differences between groups, using parametric and non parametric tests as appropriate. In order to better characterize group differences, six variables (gender, asymmetry at onset, smoking, age at onset, disease duration, levodopa latency) were analyzed in a multivariate context through multivariate logistic regression, with the presence of *LRRK2* mutations as dichotomous response variable. Furthermore, we compared motor phenotype and dichotomous values of selected UPDRS items. Statistical analyses were conducted using R statistical software (see Supplementary material).

## 3 Results

### 3.1 Mutation prevalence

Among the 2523 unrelated consecutive PD patients, 40 (1.6%) resulted to be carriers of *LRRK2* mutations, G2019S being the most frequent (1.35%; Table 1). No mutation was found in all the other patients with alternative clinical diagnoses (*N* = 453). *LRRK2* mutations were significantly more frequent in familial than in sporadic PD cases (Table e-2). In all G2019S carriers, genotypes were compatible with the common haplotype (haplotype n.1).

**Table 1:** *LRRK2* mutations identified in PD patients.

	N of PD patients	Mutation	N carriers (%)
Exon 41	2523	G2019S	34 (1.35%)^a^
		I2020L	1 (0.04%)
Exon 31	1088	R1441C	4 (0.37%)^b^
		R1441H	1 (0.09%)
Total	2523	All	40 (1.59%)

a One patient was homozygote for G2019S, and one was carrier of the *GBA*-N370S mutation.

b One patient was carrier of the *GBA*-N370S mutation.

We found several cases with rare genotypes (detailed case reports available as Supplementary material). Notably, one patient was carrier of the novel I2020L missense variant. The I2020 residue is involved in a well-known mutation (I2020T), and several in silico tools (PolyPhen, SIFT, Mutation Taster) predict that the I2020L change is damaging (Table e-3). Therefore, we considered this new variant as a mutation. Finally, we disclosed two synonymous variants in the heterozygous state: c.6054C > T (p.Y2018Y), which has already been described [2], and the novel c.6021C > T (p.P2007P) variant.

### 3.2 Clinical features and gender effect

A total of 49 PD *LRRK2* carriers were included in the analysis of clinical features, resulting from the sum of *N* = 40 (24F/16M) from the case series and *N* = 10 (6M/4F) affected living relatives, excluding *N* = 1 patient with clinical diagnosis of PD whose *post-mortem* examination revealed a PSP-like tauopathy (see Supplementary material). Demographic and clinical features of *LRRK2*-carriers did not differ from those of non-carriers (Table 2). Gender distribution resulted to be the only differential feature between the two groups, as most of the carriers were female (57% *vs*. 40%, *p* = 0.0101). This difference remained significant after excluding the 10 living relatives recruited in addition to the case series (60% *vs*. 40%, *p* = 0.015). After adjusting for disease duration and age, the frequency and severity of major motor and non-motor symptoms were similar.

**Table 2:** Motor clinical features of PD patients according to genetic status (*LRRK2*-carriers *vs*. non-carriers) and gender.

	*LRRK2*-carrier	Non-carrier		*LRRK2*-carrier female	*LRRK2*-carrier male	Non-carrier female	Non-carrier male
**Demographic and clinical variables**	*N* = 49	*N* = 2343	*p* value^c^	*N* = 28	*N* = 21	*N* = 947	*N* = 1396
Female gender, n (%)	28 (57%)	947 (40%)	0.0101				
Age at onset	55.82 ± 11	56.37 ± 10	NS	56.39 ± 12.14	55.05 ± 11.50	57.13 ± 10.64	55.84 ± 10.59
Disease duration	12.33 ± 6.50	11.64 ± 6.56	NS	12.50 ± 7.01	12.10 ± 5.92	11.82 ± 6.74	11.52 ± 6.43
Levodopa latency^a^	1.93 ± 1.83	2.63 ± 2.82	NS	1.85 ± 1.75	2.05 ± 1.99	2.65 ± 3.06	2.61 ± 2.65
Asymmetry at onset, n/total (%)	46/49 (94%)	1975/2281 (87%)	NS	26/28 (93%)	20/21 (95%)	791/919 (86%)	1184/1362 (87%)
Never-smoker^b^, *n*/total (%)	29/49 (59%)	1294/2037 (64%)	NS	21/28 (75%)^e^	8/21 (38%)^e^	642/802 (80%)	652/1235 (53%)
**UPDRS on**	*N* = 48	*N* = 2101	*p* value^d^	*N* = 28	*N* = 20	*N* = 858	*N* = 1243
Age at UPDRS assessment	67.29 ± 10.21	67.59 ± 10.18	NS	68.11 ± 9.69	66.15 ± 11.05	68.44 ± 10.22	67.00 ± 10.12
Disease duration at UPDRS assessment	11.79 ± 6.60	11.20 ± 6.61	NS	11.93 ± 6.97	11.60 ± 6.21	11.35 ± 6.75	11.10 ± 6.51
Part I – Mentation	2.79 ± 2.75	3.03 ± 2.42	NS	2.92 ± 2.59	2.59 ± 2.18	3.00 ± 2.62	3.05 ± 2.73
Part II – ADL	13.95 ± 8.14	13.13 ± 7.33	NS	13.81 ± 8.70	14.18 ± 7.46	12.80 ± 7.45	13.34 ± 7.25
Part III – Motor score	24.69 ± 14.74	24.01 ± 12.92	NS	25.57 ± 16.32	23.45 ± 12.52	24.04 ± 13.53	24.00 ± 12.49
Motor Phenotype PIGD-IND-TD, %	81-7-12	76-7-17	NS	88-4-8	70-12-18	80-4-16	78-4-18
**Hoehn/Yahr Stage**	2.56 ± 0.96	2.5 ± 0.87	NS	2.70 ± 1.04	2.38 ± 0.83	2.57 ± 0.92	2.45 ± 8.83
**UPDRS off**	*N* = 33	*N* = 591	*p* value^d^	*N* = 20	*N* = 13	*N* = 226	*N* = 365
Age at UPDRS assessment	64.48 ± 10.38	66.99 ± 9.61	NS	65.15 ± 10.06	63.46 ± 11.21	68.21 ± 9.65	66.25 ± 9.53
Disease duration at UPDRS assessment	12.36 ± 6.84	11.47 ± 6.94	NS	12.30 ± 6.37	12.46 ± 7.77	11.53 ± 7.62	11.44 ± 6.50
Part II – ADL	20.11 ± 8.07	15.99 ± 8.18	0.0448	20.53 ± 6.84	19.45 ± 10.01	15.42 ± 8.87	16.34 ± 7.72
Part III – Motor score	34.52 ± 13.51	31.82 ± 13.90	NS	33.80 ± 13.18	35.62 ± 14.47	31.54 ± 15.44	31.99 ± 12.90
Motor Phenotype PIGD-IND-TD, %	82-4-14	71-8-21	NS	88-4-4	72-0-28	72-9-19	70-8-22

Abbreviations: ADL, activities of daily living; IND, indeterminate; PIGD, postural instability and gait difficulty; PD, Parkinson's Disease; TD, tremor-dominant; UPDRS, Unified Parkinson's Disease Rating Scale.

Values are expressed as mean ± standard deviation, unless different parameters are specified.

a Time elapsed (years) from PD onset to initiation of levodopa therapy.

b Data are based on patient self-reporting. Current and former smokers were aggregated into the single category of smokers.

c Multivariate analysis.

d Age at UPDRS assessment and disease duration were always included in the analysis of other variables.

e Female never-smokers were significantly more common than male never-smokers among LRRK2-carriers (*p* = 0.0323). No other significant differences were present between carrier females and carrier males, and versus the respective non-carrier group.

We compared carrier females to carrier males, and against the respective non-carrier group (Table 2). We did not find any significant difference, with exception of smoking (more common in males in both groups). Major milestones of PD progression (falls, postural instability, freezing of gait, dysphagia and speech difficulties) did not show any significant gender-related difference, both in the Off- and in the On-state (data not shown).

In an attempt to evaluate the genetic component for PD in males and females regardless of genetic status, we compared family history in all PD cases (*N* = 2523). Women reported a family history of parkinsonism more frequently than men, but the difference was statistically significant only considering all relatives up to 3rd degree relatives (*p* = 0.017). However, a clear trend was evident also in 1st degree relatives (*p* = 0.058; Supplementary Table e-4).

## 4 Discussion

To the best of our knowledge, this is the largest consecutive series of patients with primary parkinsonism collected at a single clinical referral centre and tested systematically for major *LRRK2* mutations. Our findings confirm that G2019S is the most common mutation in the Italian PD population, while it is virtually absent in patients with other primary parkinsonian syndromes. Critically, it is more common in women.

Large epidemiological studies have consistently reported a higher incidence (approx. 1.5 times) of sporadic idiopathic PD in men than in women [8]. Although the basis of this difference has not been clarified so far, it has been suggested that the predominance in males is due to more frequent occupational or recreational exposure to toxins, but a putative neuroprotective role played by estrogens has also been suggested [9]. According to Mendelian inheritance, autosomal genetic forms of PD should follow an even distribution of gender among affected cases. However, *LRRK2* mutations have a low life-time penetrance, approximately 30–40% [1]. Therefore, PD in *LRRK2* carriers should still be considered of multifactorial etiology, the intervention of other genetic and/or environmental factors being mandatory for the development of disease. Accordingly, we would expect a similar 60:40 male-to-female distribution. However, this is not the case, as we found a surprising overturn leading to 57% prevalence in *LRRK2* female carriers.

A relatively higher percentage of women amongst *LRRK2*-carriers compared to patients with idiopathic-PD has previously been reported, but only in the Ashkenazi Jewish population [2], [3], [4]. Several other studies have reported that *LRRK2* carriers were mainly female, but the difference between genders did not reach statistical significance, probably because of small sample size [e.g. Ref. [10]. If *LRRK2*-carrier women have a greater load than men, women might be expected to develop disease earlier or progress faster than men, or both. A recent study on a large *LRRK2* PD cohort confirmed the former hypothesis, demonstrating that onset of disease occurs 5 years earlier in women [11]. On the other hand, our extensive analysis did not reveal any gender-related difference in clinical phenotype, including motor and non-motor symptom severity and measures of disease progression.

In this scenario, one could set forth the hypothesis that this gender-related effect applies not only to *LRRK2* and to other genetic forms of PD [5], but also to sporadic idiopathic PD. To further explore this hypothesis, we additionally investigated the family history of PD in the whole cohort of unrelated consecutive PD patients according to gender. The rationale of this analysis followed what is usually described in other complex multifactorial inherited diseases: when subjects of the less commonly affected gender manifest disease, their relatives are at increased risk because of the relatively larger ‘genetic load’ overcoming protective factors [12]. Accordingly, we found that PD is more common in the family history of women than men, regardless of *LRRK2* status (Table e-4).

Alternatively, it could be speculated that the LRRK2 mutated protein may interact with specific female hormonal or genetic factors, thus potentially explaining the 57% of female *LRRK2*-carriers, even exceeding the expected 50% rate in the presence of autosomal distribution of the mutation. Our analysis of clinical features confirmed that female carriers do not have more severe symptoms than male carriers or female non-carriers and their symptoms do not differ in any way. Their age at onset is similar and their disease does not appear to progress more rapidly. Hence, interaction between *LRRK2* and specific female factors does not seem likely.

Our data support the hypothesis that *LRRK2*-associated PD phenotype is not distinguishable from idiopathic PD, as we did not find any remarkable clinical difference between *LRRK2*-carriers and non-carriers. The missense variant I2020L, which we found in one of our patients, involved the same residue of a well-known mutation, I2020T [e-6]. Despite in silico predictions suggesting a damaging effect, further studies including co-segregation analysis and functional assays are necessary to include the I2020L variant among *LRRK2* pathogenic mutations.

Several strengths of our study are worth mentioning. First, *LRRK2*-carriers were identified within a large consecutive series of patients devoid of bias due to pre-selection based on clinical or demographic features, such as ethnicity, age at onset or family history. Second, the consecutive enrollment from a single tertiary centre enabled an exhaustive and standardized clinical assessment in all cases. Finally, this is the first study investigating possible gender effects on a Caucasian non-Jewish population, including not only distribution, but also a comprehensive investigation of potential differences in motor and non-motor clinical features.

In conclusion, we confirm a higher frequency of females among *LRRK2*-carriers. This might be due to a greater genetic load compared to males, where environmental factors may play a prominent role. Further studies are required to investigate this ‘gender effect’ in larger populations, including not only *LRRK2*-carriers but also other genetic causes of PD.

## Financial disclosures relevant for the present article

None of the authors have any disclosures to report.
