# Supplementary material for: LRRK2 mutations in Parkinson's disease: Confirmation of a gender effect in the Italian population
Source: Parkinsonism Relat Disord. 2014 Aug;20(8):911–4. doi: 10.1016/j.parkreldis.2014.04.016 (PMC4144811; doi:10.1016/j.parkreldis.2014.04.016)
Supplement: Supplementary file 1 [file mmc1.doc]

**LRRK2 mutations in Parkinson’s Disease: confirmation of a gender effect in the Italian population.**

**Supplementary Material**

**Methods**

*Mutation analysis*

The mutation analysis methods adopted for the first 1,245 patients are described elsewhere.^7^ Methods for the LRRK2 analysis in the extra 1,734 patients are reported below.

The LRRK2 exon 31 and exon 41 were PCR amplified from patient DNA using these primer couples: PK8-31F: 5’-GAATGTGAGCAGGCCCAGT-3’; PK8-31R: 5’-AGAAAACCCACAATTTTAAGTG-3’; PK8-41F: 5’-TTTAAGGGACAAAGTGAGCAC-3’; PK8-41R: 5’-ACTCTGTTTTCCTTTTGACTC-3’. Mutational screening on these exons was performed using the Surveyor mutation detection kit (Transgenomic, Omaha, NE), which is based on the use of the mismatch-specific endonuclease Surveyor. Sample PCR products and wild-type amplicons are hybridized to generate heteroduplexes in the presence of mutations. Homoduplexes/heteroduplexes derived from the hybridization step are treated with the Surveyor endonuclease and the mutation detection is completed by separation of digestion fragments by agarose gel electrophoresis. DNA fragments showing mismatches were subjected to DNA sequencing using an ABI-3130XL Genetic Analyzer (Life Technologies, Foster City, CA, USA).

*Haplotype analysis*

LRRK2 haplotypes were studied in all G2019S mutation carriers. Ten LRRK2 SNP markers, spanning from exon 8 to 43, were typed by direct sequencing. The sequence analysis was performed on an ABI Prism 3130 XL Genetic Analyzer. Haplotypes were numbered in agreement to previous publications,^e-7^ see Table e-1.

**Table e-1. SNPs and haplotypes of the LRRK2 locus**

| **LRRK2 Exon** | **SNP** |  | **Haplotype 1** | **Haplotype 2** | **Haplotype 3** |
| --- | --- | --- | --- | --- | --- |
| 8 | rs732374 |  | T |  |  |
| 13 | rs28903073 |  | A | G | G |
| 34 | rs1896252 |  | C | C |  |
|  | rs1427263 |  | A | A | C |
|  | rs11176013 |  | G | G | A |
|  | rs11564148 |  | A | A | T |
|  | rs11564205 |  | A |  |  |
| 40 | rs2404834 |  | C | T | C |
| 41 | rs34637584 (p.G2019S) |  | A | A | A |
| 43 | rs10878405 |  | A | G | G |
|  | rs11176143 |  | G |  |  |

*Statistical Analysis*

Each categorical variable was analysed separately with both a t-test based on the Gaussian approximation of the proportions and the Fisher’s independence exact test. Numerical variables were analysed through a t-test or a Wilcoxon signed-rank test, where the Gaussian assumption was not fulfilled. Six variables (gender, asymmetry at onset, smoking, age at onset, disease duration, levodopa latency) were analysed in a multivariate context through multivariate logistic regression, with the presence of LRRK2 mutations as dichotomous response variable.

All the analyses concerning the UPDRS data were performed considering both medication-On and -Off status, taking into account age and disease duration at assessment. Although the values of the UPDRS test are natural numbers, in the statistical study they were considered as continuous quantities. Taking this approximation, UPDRS items (Mentation-ADL-Motor score) were studied separately through a t-test or Wilcoxon signed-rank test where the Gaussian assumption was not fulfilled. Then, age and disease duration at UPDRS were included in the multivariate analysis. These variables were considered as predictors of a linear regression model whose response variables were represented by the UPDRS subscales scores. The presence of the LRRK2 mutation was modelled as a dichotomous covariate. The analysis on the UPDRS data was completed by a Fisher’s independence exact test on the Motor phenotype (PIGD-IND-TD, defined according to established criteria^e-8^ in the Off state) and dichotomous values of selected UPDRS items.

*Additional PD-related mutations*

We tested mutations on the glucocerebrosidase (GBA) gene in all consecutive patients with Parkinsonism who participated to the DNA Biobank (manuscript in preparation). Mutations on Parkin, PINK1 and DJ1 were tested in PD patients with early onset (<40years). Mutations on the α-synuclein (SNCA) gene were tested only in familial cases, when a first-degree relative was affected.

*PSP-like Tauopathy associated G2019S mutation disclosed at post-mortem*

One patient with G2019S died recently (PD07 in ref^7^). Neuropathological analysis was consistent with PSP-like tauopathy rather than synucleinopathy, as we reported elsewhere.^e-9^ Consequently, this patient was excluded from the analysis of motor and non-motor symptoms of PD.

**Results**

**Table e-2 Distribution of LRRK2 mutations according to family history of PD.** Data are presented as number of patients (%).

|  | Patients | All LRRK2 mutations | G2019S | I2020L | Patients | R1441C | R1441H |
| --- | --- | --- | --- | --- | --- | --- | --- |
| All | 2,383 | 40 (1.68%) | 34 (1.4%) | 1 (0.04%) | 1,088 | 4 (0.37%) | 1 (0.09%) |
| Sporadic | 1,938 | 20 (1%) | 18 (0.9%) | 0 | 660 | 1 (0.15%) | 1 (0.15%) |
| Familial | 445 | 20 (4.5%) | 16 (3.6%) | 1 (0.2%) | 428 | 3 (0.70%) | 0 |
| 1^st^ degree | 255 | 16 (6.3%) | 13 (5.1%) | 1 (0.4%) | 242 | 2 (0.82%) | 0 |
| 2^nd^ degree | 122 | 1 (0.8%) | 1 (0.8%) | 0 | 120 | 0 | 0 |
| 3^rd^ degree | 68 | 3 (4.4%) | 2 (2.9%) | 0 | 66 | 1 (1.51%) | 0 |

*Evaluation of novel I2020L variant*

The novel I2020L LRRK2 mutation was analysed by in-silico tools to predict whether this amino acid substitution affects protein function. The results are reported in the Table e-3.

**Table e-3. In silico prediction on the I2020L variant.**

| In silico tool | Link | Result | Evaluation score |
| --- | --- | --- | --- |
| Polyphen | http://genetics.bwh.harvard.edu/pph2/ | Possibly damaging | 0.527^a^ |
| SIFT | http://sift.bii.a-star.edu.sg/ | Damaging | 0^b^ |
| Mutation Taster | http://www.mutationtaster.org/ | Disease causing | 0.99999^c^ |

^a^ sensitivity: 0.88; specificity: 0.90.

^b^ The SIFT output score ranges from 0 to 1. The amino acid substitution is predicted damaging if the score is <= 0.05, and tolerated if the score is > 0.05.

^c^ Probability value.

*Family History according to gender*

**Table e-4** Frequency of family history of PD in the whole cohort of 2523 unrelated consecutive PD patients.

|  | Female (1,034) | Male (1,489) | p value |
| --- | --- | --- | --- |
| 1^st^ degree | 13% | 10% | 0.058 |
| 1^st^ +2^nd^ degree | 18% | 16% | 0.112 |
| 1^st^ +2^nd^ +3^rd^ degree | 22% | 18% | **0.017** |

**Case Descriptions**

*G2019S homozygote*

This 71-year-old male subject referred to our institute at 64 years complaining about a 1-year history on left leg resting tremor. His past medical history showed hypothyroidism and hypercholesterolemia. Renal function was normal.^e-10^

Neurological examination: left-sided resting and action tremor, bradykinesia and muscle rigidity (UPDRS motor score, 10; Hoehn & Yahr stage, 1). Brain RMN was unremarkable. Dopamine transporter SPECT imaging confirmed the clinical diagnosis of PD, while fluorodeoxyglucose PET showed relative hypometabolism in the left parietal area. Treatment with rasagiline and the dopamine agonist pramipexole led to remarkable improvement. He developed insomnia and daytime sleepiness.

At 7-years’ disease duration, levodopa therapy was started with benefit. One year later, his UPDRS motor score was 18 on medications and he reported no motor fluctuations or dyskinesias. He underwent extensive neuropsychological assessment twice, (at 4- and 6-years’ disease duration) and always showed preserved cognitive functions. During the first clinical interview, he admitted he had become more impulsive and irritable after PD onset. Surprisingly, he additionally revealed that he had attempted suicide once, and that he was planning his death in the event of complete motor disability. Nonetheless, he was not clinically depressed at formal assessment. The second neuropsychological assessment disclosed the presence of computer addiction and confirmed his predisposition to dopaminergic behavioural side effects due to increased impulsivity and irritability.

Family history: one sister was affected by probable Progressive Supranuclear Palsy, with onset at 59 years of age. However, genetic analysis of LRRK2 in this sister did not reveal any pathogenic mutation (repeated twice). No other relatives were reported to have movement disorders or other neurodegenerative diseases. Most of his other siblings had normal neurological examination and some resulted to be carriers of the G2019S mutation in the heterozygous state. Neurological examination showed reduced arm swings (but no other signs of parkinsonism) in one sibling, heterozygous for the G2019S mutation.

*LRRK2-G2019S + GBA-N370S*

This 68-year-old man, resulted to be an heterozygous carrier of the LRRK2-G2019S mutation and the GBA-N370S mutation. His onset was at 49 with micrographia and resting tremor in the right lower limb. Levodopa therapy was immediately initiated with excellent motor response. He started complaining about motor fluctuations 16 years after onset. They were well compensated by the combination of pramipexole and entacapone. He was also prescribed quetiapine for night-time restlessness. At that time, he developed impulse control disorders (such as pathological gambling and hypersexuality), which resolved after pramipexole dosage reduction and psychotherapy. After one year, he started complaining about visual hallucinations, with preserved insight . At his last examination at our institute in 2013 (68-years old), he had severe on-off fluctuations with dyskinesia. His activity of daily living was reduced (UPDRS II: 14) and UPDRS motor scores were 24 (On-meds) and 40 (Off-meds). Brain MRI was unremarkable. Cognitive and behavioural assessment was performed twice (at 17- and 18-years’ disease duration, respectively) and, despite the long disease duration, he did not show signs of any cognitive dysfunction at both assessments. The behavioural assessment disclosed his past history of Impulse Control Disorders, apathy and dysphoria.

He has no family history of movement disorders. Out of three siblings, one has Alzheimer’s dementia, which started at 71 years of age.

*LRRK2-R1441C + GBA-N370S*

This patient was a 70-year-old man, carrier of LRRK2-R1441C and GBA-N370S. PD presented at the age of 44-years with micrographia and right-sided bradykinesia. After 1 year, he had good response to levodopa therapy. Three years after initiation of levodopa, he developed wearing-off and, later on, dyskinesias. At 14-years’ disease duration, he was finally referred for GPi deep brain stimulation for severe on-off motor fluctuations with good outcome. At 62 years, he had signs of mild cognitive impairment. Full-blown dementia was diagnosed 5 years later. He died at age 70 of pneumonia.

Cognitive and behavioural assessment was conducted six times, the first at the age of 59 (one year after DBS surgery) and the last at 69 (disease duration: 15 and 27 years, respectively). The first assessment revealed overall preserved cognitive functions. Three years later, he showed memory and frontal-lobe dysfunction, visual hallucinations, anxiety and sleep problems. One year later, memory and frontal-lobe functions were severely impaired with further worsening of neuropsychiatric symptoms (hallucinations, anxiety, and irritability). Finally, his severe dementia was associated with apathy, auditory and visual well-formed hallucinations along with delusions. Family history: patient was not available for a formal session of genetic counselling. He reported that his father had died at 87 years of age, with a formal diagnosis of PD at 80 years. No other relatives were reported to have had movement disorders or other neurodegenerative diseases.

*R1441H*

This 64-year old woman carries LRRK2 R1441H mutation in heterozygosis. PD presented at 59 with left-sided clumsiness, followed by resting tremor, two years later. Dopamine transporter SPECT imaging revealed reduction in the bilateral striatum, brain MRI was unremarkable. She showed good response to levodopa but did not tolerate rotigotine for left foot dystonia, which reversed to normal after its withdrawal.

Three years after levodopa initiation, wearing-off phenomenon developed presenting with off-state dystonia. She underwent an extensive neuropsychological assessment at 4-years’ disease duration (at the age of 63-years) showing normal cognitive functions and behaviour.

She has no family history of movement disorders. Her mother died at 89 years of age with dementia, which started at 80 years, probably due to widespread cerebrovascular disease.

*I2020L*

This 82-year-old woman carried the LRRK2 I2020L mutation in heterozygosis. PD presented at 60 with left limb tremor. The disease slowly progressed and levodopa therapy was started 4 years after onset. Motor fluctuations first appeared 8 years after levodopa initiation. Her medical history revealed hip replacement and liver cirrhosis She died at age 83 (disease duration: 23 years) following surgery complications (hemorroidectomy). She did not undergo extensive neuropsychological assessment, but at the last visit (age 82, disease duration: 22 years) she had no sign of cognitive decline, which is quite surprising considering the patient’s disease duration and age.

Family history: one sister, out of four siblings, who died at 92 years of age, was diagnosed with PD at approximately 70 years of age: she had resting tremor, bradykinesia, levodopa therapy with benefit, dementia in the last years. She was not available for neurological examination or genetic analysis. No other relatives were reported to have had movement disorders or other neurodegenerative diseases.

*Comments:*

In all cases, clinical features were compatible with idiopathic PD. Taken together, the three patients with more than one mutation (LRRK2-G2019S homozygote; LRKK2-G2019S + GBA-N370S; LRRK2-R1441C+GBA-N370S) did not have signs of more aggressive disease than other LRRK2-carriers and non-carriers.

**Supplementary References**

e-1. Paisán-Ruiz C, Lewis PA, Singleton AB. LRRK2: cause, risk, and mechanism. J Parkinsons Dis. 2013;3(2):85-103.

e-2. Mirelman A, Heman T, Yasinovsky K, [Thaler A](http://www.ncbi.nlm.nih.gov/pubmed?term=Thaler A%5BAuthor%5D&cauthor=true&cauthor_uid=24123150), [Gurevich T](http://www.ncbi.nlm.nih.gov/pubmed?term=Gurevich T%5BAuthor%5D&cauthor=true&cauthor_uid=24123150), [Marder K](http://www.ncbi.nlm.nih.gov/pubmed?term=Marder K%5BAuthor%5D&cauthor=true&cauthor_uid=24123150), et al. LRRK2 Ashkenazi Jewish Consortium. Fall risk and gait in Parkinson's disease: The role of the LRRK2 G2019S mutation. Mov Disord. 2013;28(12):1683-90.

e-3. Nishioka K, Kefi M, Jasinska-Myga B, [Wider C](http://www.ncbi.nlm.nih.gov/pubmed?term=Wider C%5BAuthor%5D&cauthor=true&cauthor_uid=19726410), [Vilariño-Güell C](http://www.ncbi.nlm.nih.gov/pubmed?term=Vilariño-Güell C%5BAuthor%5D&cauthor=true&cauthor_uid=19726410), [Ross OA](http://www.ncbi.nlm.nih.gov/pubmed?term=Ross OA%5BAuthor%5D&cauthor=true&cauthor_uid=19726410), et al. A comparative study of LRRK2, PINK1 and genetically undefined familial Parkinson's disease. J Neurol Neurosurg Psychiatry 2010; 81:391-395.

e-4. Healy DG, Falchi M, O'Sullivan SS, [Bonifati V](http://www.ncbi.nlm.nih.gov/pubmed?term=Bonifati V%5BAuthor%5D&cauthor=true&cauthor_uid=18539534), [Durr A](http://www.ncbi.nlm.nih.gov/pubmed?term=Durr A%5BAuthor%5D&cauthor=true&cauthor_uid=18539534), [Bressman S](http://www.ncbi.nlm.nih.gov/pubmed?term=Bressman S%5BAuthor%5D&cauthor=true&cauthor_uid=18539534), et al. Phenotype, genotype, and worldwide genetic penetrance of LRRK2-associated Parkinson's disease: a case-control study. Lancet Neurol 2008;7:583-590.

e-5. Litvan I, Bhatia KP, Burn DJ, [Goetz CG](http://www.ncbi.nlm.nih.gov/pubmed?term=Goetz CG%5BAuthor%5D&cauthor=true&cauthor_uid=12722160), [Lang AE](http://www.ncbi.nlm.nih.gov/pubmed?term=Lang AE%5BAuthor%5D&cauthor=true&cauthor_uid=12722160), [McKeith I](http://www.ncbi.nlm.nih.gov/pubmed?term=McKeith I%5BAuthor%5D&cauthor=true&cauthor_uid=12722160), et al. Movement Disorders Society Scientific Issues Committee report: SIC Task Force appraisal of clinical diagnostic criteria for Parkinsonian disorders. Mov Disord 2003;18:467-486.

e-6. Zimprich A, Biskup S, Leitner P, [Lichtner P](http://www.ncbi.nlm.nih.gov/pubmed?term=Lichtner P%5BAuthor%5D&cauthor=true&cauthor_uid=15541309), [Farrer M](http://www.ncbi.nlm.nih.gov/pubmed?term=Farrer M%5BAuthor%5D&cauthor=true&cauthor_uid=15541309), [Lincoln S](http://www.ncbi.nlm.nih.gov/pubmed?term=Lincoln S%5BAuthor%5D&cauthor=true&cauthor_uid=15541309), et al. Mutations in LRRK2 cause autosomal-dominant parkinsonism with pleomorphic pathology. Neuron 2004; 44:601-607.

e-7. [Goldwurm S](http://www.ncbi.nlm.nih.gov/pubmed?term=Goldwurm S%5BAuthor%5D&cauthor=true&cauthor_uid=16272257), [Di Fonzo A](http://www.ncbi.nlm.nih.gov/pubmed?term=Di Fonzo A%5BAuthor%5D&cauthor=true&cauthor_uid=16272257), [Simons EJ](http://www.ncbi.nlm.nih.gov/pubmed?term=Simons EJ%5BAuthor%5D&cauthor=true&cauthor_uid=16272257), [Rohé CF](http://www.ncbi.nlm.nih.gov/pubmed?term=Rohé CF%5BAuthor%5D&cauthor=true&cauthor_uid=16272257), [Zini M](http://www.ncbi.nlm.nih.gov/pubmed?term=Zini M%5BAuthor%5D&cauthor=true&cauthor_uid=16272257), [Canesi M](http://www.ncbi.nlm.nih.gov/pubmed?term=Canesi M%5BAuthor%5D&cauthor=true&cauthor_uid=16272257), et al. The G6055A (G2019S) mutation in LRRK2 is frequent in both early and late onset Parkinson's disease and originates from a common ancestor. J Med Genet. 2005;42(11):e65.

e-8 Jankovic J, McDermott M, Carter J, [Gauthier S](http://www.ncbi.nlm.nih.gov/pubmed?term=Gauthier S%5BAuthor%5D&cauthor=true&cauthor_uid=2215943), [Goetz C](http://www.ncbi.nlm.nih.gov/pubmed?term=Goetz C%5BAuthor%5D&cauthor=true&cauthor_uid=2215943), [Golbe L](http://www.ncbi.nlm.nih.gov/pubmed?term=Golbe L%5BAuthor%5D&cauthor=true&cauthor_uid=2215943), et al. Variable expression of Parkinson's disease: a base-line analysis of the DATATOP cohort. The Parkinson Study Group. Neurology 1990; 40:1529-1534.

e-9. Ruffmann C, Giaccone G, Canesi M, et al. Atypical tauopathy in a patient with LRRK2-G2019S mutation and tremor-dominant Parkinsonism. Neuropathol Appl Neurobiol 2012; 38:382-386.

e-10. [Herzig MC](http://www.ncbi.nlm.nih.gov/pubmed?term=Herzig MC%5BAuthor%5D&cauthor=true&cauthor_uid=21828077), [Kolly C](http://www.ncbi.nlm.nih.gov/pubmed?term=Kolly C%5BAuthor%5D&cauthor=true&cauthor_uid=21828077), [Persohn E](http://www.ncbi.nlm.nih.gov/pubmed?term=Persohn E%5BAuthor%5D&cauthor=true&cauthor_uid=21828077), [Theil D](http://www.ncbi.nlm.nih.gov/pubmed?term=Theil D%5BAuthor%5D&cauthor=true&cauthor_uid=21828077), [Schweizer T](http://www.ncbi.nlm.nih.gov/pubmed?term=Schweizer T%5BAuthor%5D&cauthor=true&cauthor_uid=21828077), [Hafner T](http://www.ncbi.nlm.nih.gov/pubmed?term=Hafner T%5BAuthor%5D&cauthor=true&cauthor_uid=21828077), et al. LRRK2 protein levels are determined by kinase function and are crucial for kidney and lung homeostasis in mice. [Hum Mol Genet.](http://www.ncbi.nlm.nih.gov/pubmed/21828077) 2011; 20(21):4209-23.
